# Comparative Characterization of *Vibrio cholerae* O1 from Five Sub-Saharan African Countries Using Various Phenotypic and Genotypic Techniques

**DOI:** 10.1371/journal.pone.0142989

**Published:** 2015-11-25

**Authors:** Anthony M. Smith, Berthe-Marie Njanpop-Lafourcade, Martin A. Mengel, Bradford D. Gessner, Delphine Sauvageot, Bawimodom Bidjada, Berthe N. Miwanda, Diallo M. Saliou, Adèle Kacou N’Douba, José P. Langa, Husna Ismail, Nomsa Tau, Arvinda Sooka, Karen. H. Keddy

**Affiliations:** 1 Centre for Enteric Diseases, National Institute for Communicable Diseases, Division in the National Health Laboratory Service (NHLS), Johannesburg, South Africa; 2 Faculty of Health Sciences, University of the Witwatersrand, Johannesburg, South Africa; 3 Agence de Médecine Préventive (AMP), Paris, France; 4 Institut National d’Hygiène, Lomé, Togo; 5 Institut National de Recherche Biomédicale, Kinshasa, Democratic Republic of Congo; 6 Institut National de Santé Publique, Conakry, Guinea; 7 Institut Pasteur de Côte d’Ivoire, Abidjan, Côte d’Ivoire; 8 National Institute of Health, Maputo, Mozambique; Mahidol-Oxford Tropical Medicine Research Unit, THAILAND

## Abstract

We used standardized methodologies to characterize *Vibrio cholerae* O1 isolates from Guinea, Democratic Republic of the Congo (DRC), Togo, Côte d’Ivoire and Mozambique. We investigated 257 human isolates collected in 2010 to 2013. DRC isolates serotyped O1 Inaba, while isolates from other countries serotyped O1 Ogawa. All isolates were biotype El Tor and positive for cholera toxin. All isolates showed multidrug resistance but lacked ciprofloxacin resistance. Antimicrobial susceptibility profiles of isolates varied between countries. In particular, the susceptibility profile of isolates from Mozambique (East-Africa) included resistance to ceftriaxone and was distinctly different to the susceptibility profiles of isolates from countries located in West- and Central-Africa. Molecular subtyping of isolates using pulsed-field gel electrophoresis (PFGE) analysis showed a complex relationship among isolates. Some PFGE patterns were unique to particular countries and clustered by country; while other PFGE patterns were shared by isolates from multiple countries, indicating that the same genetic lineage is present in multiple countries. Our data add to a better understanding of cholera epidemiology in Africa.

## Introduction

Cholera is well-established in Africa, with numerous outbreaks reported and tens of thousands of deaths estimated to occur annually [1]. Estimated incidence in Africa during 2000 to 2008 was 2.0–4.0 cases/1,000 population/year, more than double that reported in earlier studies in Kolkata and Haiti [2]. In 2013, Africa was the leading global source of cholera cases and deaths, with cases reported from 22 countries [3]. The 2013 case fatality rate for Africa was 2.4% and globally all five countries in 2013 reporting case fatality rates over 5% were in Africa [3]. Accurate estimates of the burden of cholera disease in Africa remain elusive [2]. To address this deficiency, the Agence de Médecine Préventive (AMP) launched the African Cholera Surveillance Network, Africhol (www.africhol.org) in 2009, funded by the Bill & Melinda Gates Foundation. The primary goals of the network are to describe cholera epidemiology more accurately, including confirmed disease burden and risk groups; construct a consortium for cholera prevention and control; and provide platforms to evaluate various public health interventions including cholera vaccine use. Africhol participating countries include: Guinea, Democratic Republic of the Congo (DRC), Togo, Côte d’Ivoire, Mozambique, Uganda, Tanzania, Cameroon, Kenya, Nigeria and Zimbabwe.

Past phenotypic characterization of *V*. *cholerae* O1 strains from sub-Saharan Africa has confirmed both serotype O1 Inaba biotype El Tor strains [4–6] and serotype O1 Ogawa biotype El Tor strains [7,8]. Resistance to antimicrobial agents is well-established in *V*. *cholerae* O1 in the region, including multidrug-resistant isolates [9–12]. The molecular basis for antimicrobial resistance in *V*. *cholerae* O1 isolates in the region is well-described, largely driven by mobile genetic elements including conjugative plasmids, transposable elements (SXT elements) and integrons [10,11,13–17]. *V*. *cholerae* O1 strains of the “altered El Tor” biotype, associated with the production of a classical type of cholera toxin (*ctxB* gene), are they are now commonplace in Africa. These have retained the fitness of their prototype O1 El Tor ancestors while acquiring enhanced virulence traits [18]. First reports of “altered El Tor” strains in sub-Saharan Africa occurred in Mozambique in 2004 [19], followed by numerous further reports from Mozambique [19,20], Zimbabwe [6], South Africa [7], Kenya [21], Zambia [22], Guinea [23] and Cameroon [24].

Molecular subtyping of *V*. *cholerae* O1 isolates from sub-Saharan Africa has been well-reviewed by De and coworkers [25]. For example: pulsed-field gel electrophoresis (PFGE) analysis was used to investigate South African outbreaks [7,11], multiple-locus variable-number tandem-repeats analysis (MLVA) showed multiple genetic lineages of among Kenyan environmental strains [26], and multi-locus sequence typing (MLST) was used to investigate the genetic lineage of Ghanaian and Mozambican isolates [17,20]. For molecular subtyping studies of bacterial pathogens in the field of public health and particularly global public health issues and interventions, it is highly recommended that standardized methodology is used to analyze bacterial pathogens. Standardized methodologies allow accurate sharing of data and inter-laboratory comparisons of molecular subtyping data. A foremost worldwide network employing such principles is PulseNet International, a molecular subtyping network for foodborne and waterborne disease surveillance (http://www.pulsenetinternational.org/). The primary bacterial subtyping technique used by PulseNet is PFGE analysis. PulseNet Africa is operational and active [27,28], with an established database of PFGE patterns for *V*. *cholerae* O1 isolated in sub-Saharan Africa [7,11,29]. Therefore, for molecular subtyping of *V*. *cholerae* O1, PFGE analysis using PulseNet methodology is currently the most functional choice for data sharing and inter-laboratory comparisons.

The ultimate genotypic method for analysis and comparison of bacterial isolates would be whole-genome sequencing (WGS) analysis. WGS of *V*. *cholerae* O1 El Tor strains from sub-Saharan Africa have shown clonal relatedness to O1 El Tor strains isolated elsewhere in the world. Recent WGS studies have included the following: Analysis of 154 whole-genome sequences from globally representative strains has shown how the seventh cholera pandemic spread from the Bay of Bengal in three independent but overlapping waves with a common ancestor in the 1950s, with data suggesting that transmission of cholera to South America may have been via Africa [30]. From Kenya, clinical and environmental strains have been shown to map onto wave three of the monophyletic seventh pandemic *V*. *cholerae* O1 El Tor phylogeny; notably some environmental strains were also identified which were phylogenetically distinct from the main O1 El Tor lineage [4]. From Mozambique, strains have been shown to belong to the phylocore group 1 clade of *V*. *cholerae*, which includes the seventh pandemic O1 El Tor [18].

Reviews of epidemiological data have identified several major foci of recurrent cholera epidemics in the interior [31] and along the coasts [32] of the African continent. However, there is currently a paucity of comparable microbiological data to support these observations. The sustained sampling using a combination of standardized microbiological and molecular epidemiological methods would determine whether these foci are connected thereby optimizing intervention strategies and mitigating cholera transmission.

In the current study, we employed standardized methodologies to phenotypically and genotypically characterize, compare and investigate *V*. *cholerae* O1 isolates from Guinea, DRC, Togo, Côte d’Ivoire and Mozambique collected in 2010 to 2013, to provide a basis for understanding disease spread and the relatedness of circulating *V*. *cholerae* O1 isolates in sub-Saharan Africa. This information may additionally highlight the emergence of new predominant multidrug-resistant or virulent clones, as well as provide baseline data for future comparisons, should new cholera cases emerge in Africa or on other continents.

## Materials and Methods

### Africhol and identification of cholera cases

Africhol has implemented case-based cholera surveillance in dedicated zones of high-incidence countries in Africa including: Guinea, DRC, Togo, Côte d’Ivoire, Mozambique and Uganda. Methods for identification of cases in fixed surveillance zones and during outbreaks outside of surveillance zones are described elsewhere (D. Sauvageot, manuscript in preparation). Briefly, all participating sites in the network identified suspect cholera cases through passive detection at health facilities level. We used the WHO case definition for suspect and confirmed cases adapted to include patients older than two years of age. (http://www.who.int/topics/cholera/surveillance/en/). For cases in Guinea, the mean age of patients was 29.94 years with a median of 26 years; 50% of patients were female and 50% of patients were male. For cases in DRC, the mean age of patients was 19.19 years with a median of 12 years; 52% of patients were female and 48% of patients were male. For cases in Togo, the mean age of patients was 28.59 years with a median of 27 years; 48% of patients were female and 52% of patients were male. For cases in Côte d’Ivoire, the mean age of patients was 28.92 years with a median of 27 years; 39% of patients were female and 61% of patients were male. For cases in Mozambique, the mean age of patients was 22.71 years with a median of 20 years; 47% of patients were female and 53% of patients were male. Stool culture was performed systematically for all the suspect cholera cases attending the cholera treatment centers. In case of large outbreaks and limited staff capacity, culture was performed on a selected number of cases. In all settings, a confirmed case was defined as the isolation and identification of *V*. *cholerae* O1 or O139 from a stool sample or rectal swab specimen. Epidemiological, clinical, behavioral, biological and environmental information was collected using standardized case report forms. Surveillance activities continued for at least one full year following the starting date of the project in each country. *V*. *cholerae* isolates investigated for this work were collected from Africhol surveillance zones and outbreak sites from 2010 to 2013. In Guinea, isolates derived from the 2012 epidemic in the prefectures of Boffa, Forecariah Mamou and Conakry. In Côte d’Ivoire, isolates derived from the 2012 outbreak in the Adiake district and Abidjan city. In Togo, isolates derived from outbreaks in the Zio prefecture (2011), lac regions (2012) and Lome city [districts 1–4 and Golfe] (2010, 2011, and 2012). In DRC, isolates derived from three outbreaks in 2011 in the Oriental province, Equator province and Bandundu Province. In Mozambique, isolates derived from epidemics during 2012 and 2013 in Sofala Province, Cabo Delgado and Nampula.

### Ethical approval

Africhol provides technical and financial resources to national Ministries of Health to support cholera surveillance in affected countries. In these countries, cholera is part of the national public health surveillance through the integrated disease surveillance and response system supported by WHO-AFRO. In each country, we reviewed the support available through Africhol and requested the Ministry of Health to make a determination as to whether they viewed the activities as research or part of routine public health surveillance, and thus whether ethical review board approval was needed or not. The Africhol study was approved by the Ministries of Health of each participating country. The enhanced surveillance conducted by the country teams was covered by the respective national public-health law as an integral part of the public-health mandate of the ministry of health and its subordinate executing agency. Therefore, the Ministries sought no separate approval from an institutional review board.

Regardless of the country determination of ethical review board requirements, participants provided their verbal consent to the health worker collecting the information and stool sample. If the patient was a minor/child, verbal consent was obtained from the next of kin, caretakers or guardians on behalf of the minor/children. This was documented on the surveillance form. Ministry of Health authorities approved of all activities supported by Africhol, including the consent procedure.

Within the cholera-endemic countries, those authors working with the data had access to patient information, as cholera is a notifiable condition and countries need this information to initiate a public health response as prescribed by the WHO as part of International Health Regulations. All other authors not working within the cholera-endemic countries had no access to identifying patient information at any time, i.e., data was analyzed anonymously by these authors.

### Extended characterization of *V*. *cholerae* O1 isolates

Isolates collected during this study were forwarded to the Centre for Enteric Diseases (CED), National Institute for Communicable Diseases (NICD), South Africa. The CED confirmed the identification of *V*. *cholerae* O1 and performed extended characterization of the isolates.

### Phenotypic characterization of isolates

Bacteria were received in Stock Culture Agar (Bio-Rad Laboratories, Marnes-la-Coquette, France) and sub-cultured onto 5% Blood Agar (Diagnostic Media Products, National Health Laboratory Service, South Africa) and Thiosulfate Citrate Bile Salts Sucrose Agar (Diagnostic Media Products), to check for viability and purity. Cultures were identified using standard phenotypic microbiological identification and serotyping techniques, briefly described as follows. As required, bacterial colonies were identified using the VITEK-2 COMPACT 15 automated microbial identification system (bioMérieux, Marcy-l'Étoile, France). Serogrouping and serotyping was determined by the slide agglutination method with polyvalent antisera and mono-specific Inaba and Ogawa antisera (Mast Diagnostics, Merseyside, United Kingdom). Antimicrobial susceptibility testing was performed on Mueller-Hinton Agar (Diagnostic Media Products) using the Etest method (bioMérieux); the following antimicrobials were tested: ampicillin, ceftriaxone, cotrimoxazole, chloramphenicol, nalidixic acid, ciprofloxacin, tetracycline, erythromycin and nitrofurantoin as an alternative to furazolidone. Minimum inhibitory concentration (MIC) breakpoints for determination of antimicrobial resistance are shown in Table 1; these were interpreted in accordance with the Clinical and Laboratory Standards Institute (CLSI) [33] with the exception of erythromycin which was interpreted according to breakpoints described by Ng and coworkers [34].

**Table 1:** Summary of phenotypic data for *V*. *cholerae* O1 isolates.

Country	Number of isolates investigated	Year of isolation	Serotype	Biotype	Percentage of isolates showing resistance to antimicrobial agents *								
					Amp	Cro	Cot	Chl	Nal	Cip	Tet	Ery	Nit
Guinea	125	2012	O1 Ogawa	El Tor	0.7	0	98.5	5.9	0	0	0	0	60.3
DR Congo	36	2011	O1 Inaba	El Tor	0	0	97.4	0	18.4	0	0	5.3	71.1
Togo	42	2010–2012	O1 Ogawa	El Tor	0	0	100	0	90.5	0	0	0	71.4
Ivory Coast	28	2012	O1 Ogawa	El Tor	0	0	96.6	10.3	100	0	0	0	93.1
Mozambique	26	2012–2013	O1 Ogawa	El Tor	100	100	100	96.3	100	0	48.1	100	92.6

* Isolates were determined to be resistant to antimicrobial agents at the following MIC breakpoints: ampicillin (Amp), ≥16 μg/ml; ceftriaxone (Cro), ≥2 μg/ml; ≥16 μg/ml; cotrimoxazole (Cot), ≥4 μg/ml; chloramphenicol (Chl), ≥16 μg/ml; nalidixic acid (Nal), ≥32 μg/ml; ciprofloxacin (Cip), ≥2 μg/ml; tetracycline (Tet), ≥8 μg/ml; erythromycin (Ery), ≥4 μg/ml; nitrofurantoin (Nit), ≥64 μg/ml.

### Genotypic characterization of isolates

Conventional PCR and analysis of PCR products using agarose gel electrophoresis was used to detect the presence of allelic variants of the toxin co-regulated pilus (*tcpA* gene) which determined the biotype (classical or El Tor) of *V*. *cholerae* O1 isolates [35]. Real-time PCR using an Applied Biosystems 7500 instrument equipment (Life Technologies, Foster City, CA) was used to detect for the presence of cholera toxin (*ctxA* gene) [36]. Molecular subtyping of isolates was performed using pulsed-field gel electrophoresis (PFGE) analysis. For PFGE, analysis of *Not*I digested genomic DNA was performed with a Bio-Rad CHEF-DR III electrophoresis system (Bio-Rad Laboratories, Hercules, USA) using a PulseNet protocol [37]. PFGE patterns were analyzed using BioNumerics (version 6.5) Software (Applied Maths, Sint-Martens-Latem, Belgium) with dendrograms of the patterns created using the unweighted pair group method with arithmetic averages (UPGMA), with analysis of banding patterns incorporating the Dice-coefficient at an optimization setting of 1.5% and a position tolerance setting of 1.5%.

## Results

**Fig 1:**
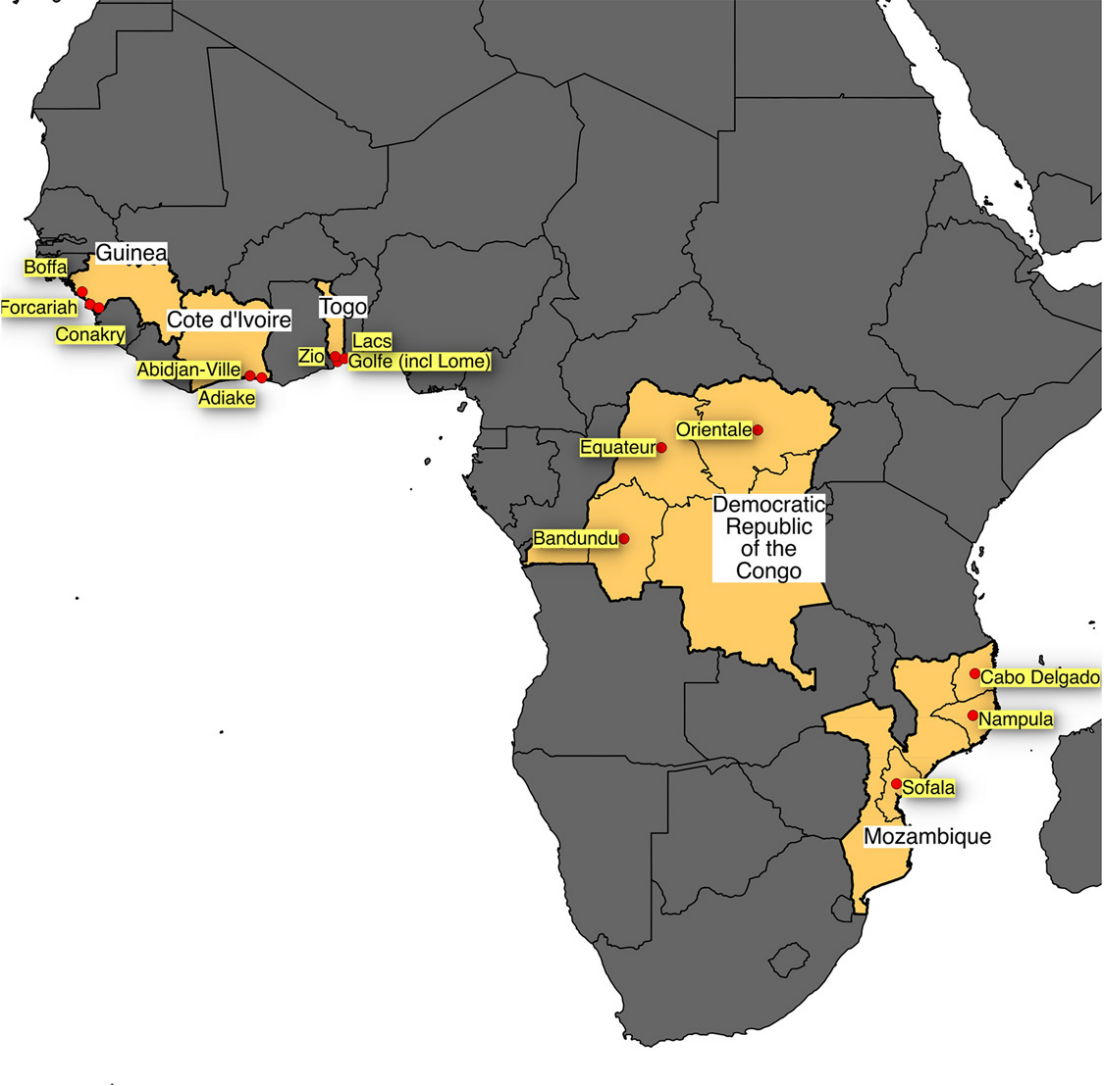
Map of Africa showing the countries described in the current study.

We investigated a total of 257 *V*. *cholerae* O1 isolates from cholera patients from five sub-Saharan African countries (Fig 1) isolated during 2010 to 2013 (Table 1); Guinea (n = 125), DRC (n = 36), Togo (n = 42), Côte d’Ivoire (n = 28) and Mozambique (n = 26). Isolates from the DRC (year 2011) were *V*. *cholerae* O1 Inaba, while isolates from other countries were *V*. *cholerae* O1 Ogawa. All isolates were identified as the El Tor biotype and all were positive for the presence of cholera toxin (*ctxA* gene).

Results of antimicrobial resistance of isolates are summarized in Table 1. Briefly, tetracycline resistance was not detected in isolates from Guinea, DRC, Togo and Côte d’Ivoire; while 48.1% Mozambican isolates were resistant to tetracycline. No isolates tested were resistant to ciprofloxacin; but nalidixic acid resistance ranged from 18.4% in DRC to 90.5% in Togo and 100% in Côte d’Ivoire and Mozambique. In Mozambique, ceftriaxone resistance occurred in 100% of isolates; whereas isolates from Guinea, DRC, Togo and Côte d’Ivoire showed no resistance to ceftriaxone.

**Fig 2:**
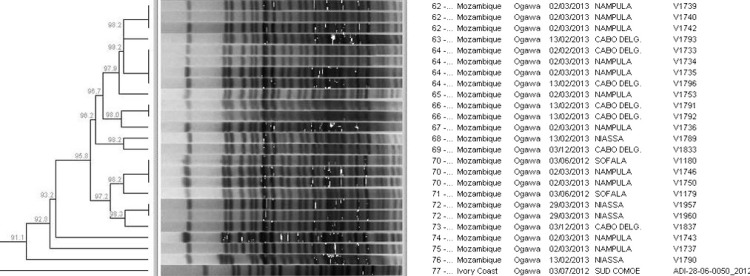
Snapshot of dendrogram of PFGE (*Not*I digestion) patterns for *V*. *cholerae* O1 isolates.

Dendrogram analysis of PFGE patterns from the 257 *V*. *cholerae* O1 isolates showed 77 PFGE patterns (S1 Fig). The number of isolates and number of PFGE patterns by country were, respectively, 42 and 6 in Togo, 36 and 11 in DRC, 28 and 12 in Côte d’Ivoire, 26 and 17 in Mozambique, and 125 and 39 in Guinea. Some PFGE patterns were unique to particular countries and clustered by country. Isolates from Mozambique for example, with the exception of two, clustered together (24 isolates) with an overall PFGE pattern similarity of 91.1% (cluster 1; S1 Fig & Fig 2): this Mozambique subtype cluster was distinct and appeared unrelated to subtypes from the other four countries. Similarly, most PFGE patterns from Guinea were unique to Guinea and clustered together (83 isolates) with an overall PFGE pattern similarity of 95.3% (cluster 2; S1 Fig), and most PFGE patterns from DRC were unique to that country clustering together (22 isolates) with an overall PFGE pattern similarity of 97.6% (cluster 3; S1 Fig).

**Fig 3:**
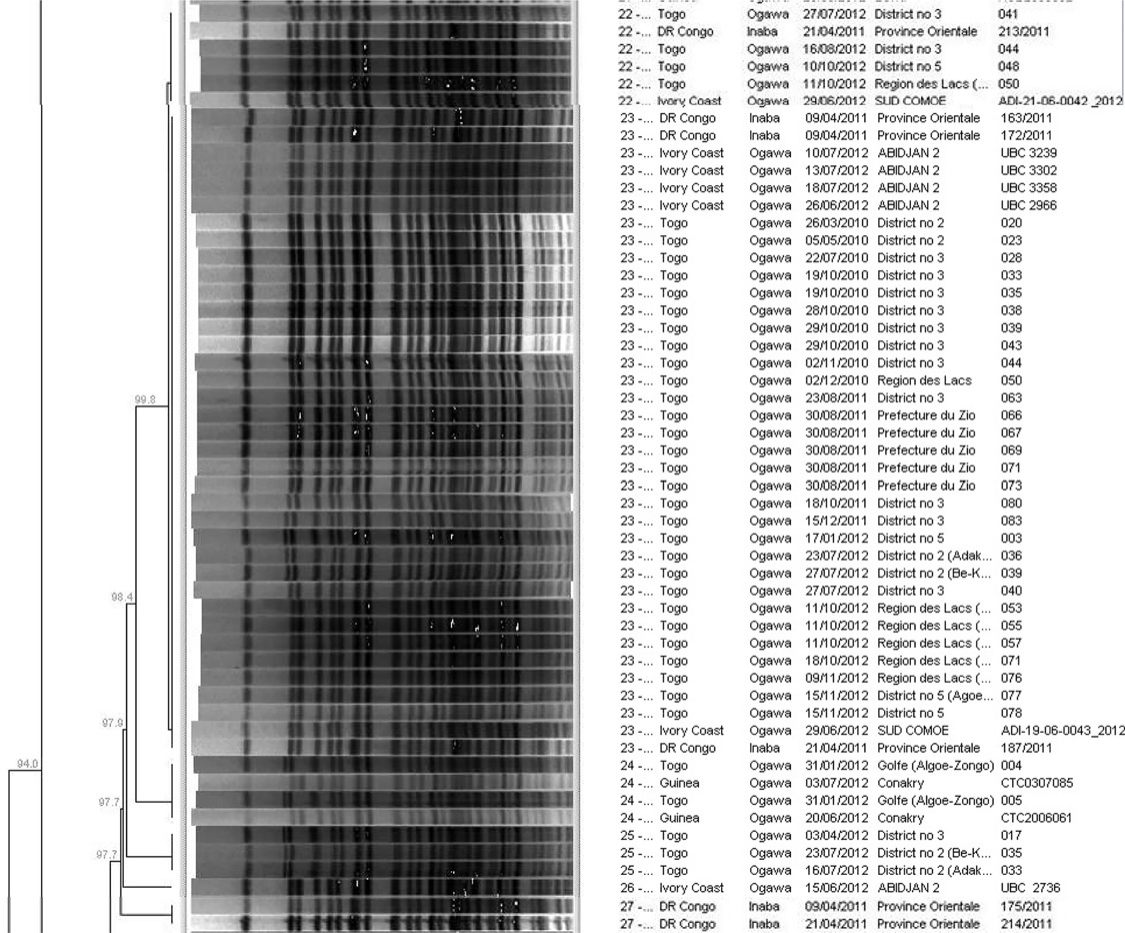
Snapshot of dendrogram of PFGE (*Not*I digestion) patterns for *V*. *cholerae* O1 isolates.

The dendrogram of PFGE patterns from all countries also showed that certain PFGE patterns were shared by isolates from multiple countries, demonstrating a relationship between isolates across a wide area: a cluster of isolates from Togo, DRC, Côte d’Ivoire and Guinea, shared a similar PFGE pattern and clustered together (53 isolates) with an overall PFGE pattern similarity of 97.7% (cluster 4; S1 Fig & Fig 3); a second cluster of isolates from DRC, Côte d’Ivoire and Guinea, shared a similar PFGE pattern and cluster together (40 isolates) with an overall PFGE pattern similarity of 97.7% (cluster 5; S1 Fig). Interestingly, a cluster comprising six isolates from Côte d’Ivoire and one isolate from Mozambique (cluster 6; S1 Fig), displayed the PFGE pattern for the Haiti cholera outbreak strain [24], a PFGE pattern previously seen in both South Africa [7] and Cameroon [24].

## Discussion

We employed standardized methodology to phenotypically and genotypically investigate and characterize *V*. *cholerae* O1 isolated during 2010 to 2013 from Guinea, DRC, Togo, Côte d’Ivoire and Mozambique. We investigated 257 *V*. *cholerae* O1 human isolates. To the best of our knowledge this is the most comprehensive standardized analysis of *V*. *cholerae* isolates from Africa to date. Both *V*. *cholerae* O1 Inaba and *V*. *cholerae* O1 Ogawa were identified (Table 1). All isolates were identified as the El Tor biotype, which was not surprising, as the classical biotype has never been reported from Africa, nor has the classical biotype been reported in recent years from other countries worldwide [38]. All isolates were positive for the presence of cholera toxin (*ctxA* gene), the major virulence determinant of the bacterium, and characteristic of almost all O1 serotypes [39,40].

*V*. *cholerae* O1 isolates from all countries showed resistance to multiple antimicrobial agents (Table 1). Isolates from DRC, Togo and Côte d’Ivoire showed similar antimicrobial susceptibility profiles, including resistance to cotrimoxazole, nalidixic acid and nitrofurantoin. As compared to these three countries, isolates from Guinea showed similar antimicrobial susceptibility profiles, except that isolates from Guinea showed no nalidixic acid resistance. In all likelihood, the molecular basis for antimicrobial (excluding nalidixic acid) resistance in the isolates described above, could be ascribed to the SXT element, a self-transmissible chromosomally integrating genetic element that can carry genes conferring resistance to multiple antimicrobial agents, including streptomycin, sulfamethoxazole, trimethoprim, furazolidone and chloramphenicol [10,13,15,16].

In contrast to the four countries described above, isolates from Mozambique showed resistance to cotrimoxazole, chloramphenicol, nalidixic acid, tetracycline, erythromycin, nitrofurantoin, ampicillin and ceftriaxone. Resistance to ceftriaxone strongly suggests the presence of extended-spectrum β-lactamase (ESBL) activity, albeit still a rare trait in this species of bacteria. We hypothesize the presence of ESBL activity such as that conferred by a plasmid-borne TEM-63 ESBL may be responsible, as has been previously reported from South Africa on the Mozambican border [11]. We did not test for the presence of ESBL activity in these isolates, as cephalosporins are not recommended for treatment of cholera. Should investigators want to test for ESBL activity, an appropriate phenotypic method would be the combination disk diffusion-based screening test, as previously described by Ismail and coworkers [7]. The antimicrobial susceptibility profile of Mozambique isolates appears to have been established and evolving from as far back as 1997, as isolates from a Mozambique epidemic in 1997 to 1998 have showed resistance to ampicillin, chloramphenicol, erythromycin, nalidixic acid and tetracycline [41].

Rehydration therapies remain the mainstay of cholera treatment, however should antimicrobial treatment of patients be needed; the WHO lists doxycycline and ciprofloxacin as options for antimicrobial management of cholera. The variety of antimicrobial resistance patterns found in our sample of *V*. *cholerae* O1 isolates indicates a need to monitor these resistance patterns locally and continuously adapt the treatment recommendations accordingly. This will help maintain the already limited treatment options in the region and inform the regulation of the current poorly regulated pharmaceutical market.

For the five countries investigated; dendrogram analysis of PFGE patterns from the 257 *V*. *cholerae* O1 isolates showed 77 PFGE patterns (S1 Fig). Some PFGE patterns were unique to particular countries and clustered by country, while other PFGE patterns were shared by isolates from multiple countries. All Mozambique isolates (with the exception of two) clustered together (24 isolates) with an overall PFGE pattern similarity of 91.1% (cluster 1; S1 Fig & Fig 2). This Mozambique subtype cluster was distinct and appeared unrelated to subtypes from the other four countries, a finding which complements the different antimicrobial susceptibility profile of Mozambique. Mozambique isolates also had the highest PFGE pattern diversity index (total number of patterns/total number of isolates) among all countries, indicating that Mozambique isolates have the highest genetic diversity among evaluated countries. Similar to Mozambique, most Guinea and DRC isolates had PFGE patterns unique to the country.

Some PFGE patterns were shared by isolates from multiple countries and collected during different study years; this indicated a relationship between isolates from different countries, i.e. indicating that the same genetic lineage is present in different countries. For example: a cluster of isolates from Togo, DRC, Côte d’Ivoire and Guinea, shared a similar PFGE pattern and clustered together (53 isolates) with an overall PFGE pattern similarity of 97.7% (cluster 4; S1 Fig & Fig 3). This cluster included most isolates from Togo (38 out of 42 isolates) collected from five districts in 2010 to 2012, isolates collected in 2011 (one site in DRC) and isolates collected in 2012 (two sites in Côte d’Ivoire and one site in Guinea). Togo isolates also showed the lowest PFGE pattern diversity index (total number of patterns/total number of isolates) among all countries, indicating that among all countries, Togo isolates were the most clonal. Another example of clustering of isolates from multiple countries was a cluster which represented DRC, Côte d’Ivoire and Guinea, which shared a similar PFGE pattern and clustered together (40 isolates) with an overall PFGE pattern similarity of 97.7% (cluster 5; S1 Fig). This cluster included isolates collected in 2011 (three sites in DRC) and isolates collected in 2012 (three sites in Côte d’Ivoire and one site in Guinea). Similarities between PFGE patterns from multiple countries may be the result of the population movements in the West-Africa region where people (traders, fishermen, students, etc.) move frequently and easily from one country to another. Recent studies have highlighted the possible contibution of marginalised migrant populations in Africa to the spread of cholera on a regional level [42]. Interestingly, a cluster comprising six isolates collected from Côte d’Ivoire in 2012 and one isolate collected from Mozambique in 2013 (cluster 6; S1 Fig), displayed the PFGE pattern for the Haiti cholera outbreak strain, a PFGE pattern previously seen in both South Africa [7] and Cameroon [24]. It is possible that travel links between African countries has resulted in similar strains in different endemic settings, but more data are needed to clarify the links between the strains found in Nepal and Africa and how they fit into the global population of *V*. *cholerae*. All the analysis and discussion described above was conducted following dendrogram analysis of patterns using the UPGMA method (already outlined in the materials and methods section). When dendrogram analysis was performed using an alternative Neighbour-Joining method, the overall clustering of isolates was very similar (data not shown). This suggested that the UPGMA method and the Neighbour-Joining method were both acceptable methods for cluster analysis of PFGE patterns.

Limitations of our study include the following. We have not yet investigated virulence determinants by DNA sequencing, therefore no data are available describing the allelic variants of *ctxA*, *ctxB*, *tcpA* and *rstR* genes among isolates; these genes can be useful markers to characterize CTXΦ phage types and track *V*. *cholerae* O1 isolates. Therefore we do not know whether any isolates were of the “altered El Tor” biotype associated with the production of a classical type of cholera toxin. We also have not yet investigated the molecular basis for antimicrobial resistance, nor did we investigate the presence of mobile genetic elements (plasmids, transposons, SXT-elements and integrons), which could potentially house antimicrobial resistance genes and mediate transfer of antimicrobial resistance. These evaluations will be conducted and reported in a follow up study involving the WGS analysis of selected *V*. *cholerae* O1 isolates. WGS will particularly assist to provide a phylogenic context among the *V*. *cholerae* O1 isolates, as PFGE analysis has limited ability and mostly gives information on relatedness of PFGE patterns to assist in tracking the spread of isolates.

In conclusion, a better understanding of the molecular epidemiology of cholera in Africa may contribute to the prevention and control of outbreaks through comprehensive, integrated regional strategies, including targeted vaccine campaigns based on scientific evidence. Molecular subtyping of isolates using PFGE analysis highlighted a complex relationship among *V*. *cholerae* O1 isolates in Africa. Some PFGE patterns were unique to particular countries and clustered by country; while other PFGE patterns were shared by isolates from multiple countries indicating that the same genetic lineage is present in multiple countries. Also noted, were the varied antimicrobial susceptibility profiles of *V*. *cholerae* O1 between countries; in particular, the susceptibility profile of isolates from Mozambique (East-Africa) was distinctly different from the profiles of isolates from other countries located in West- and Central-Africa. New tools for cholera control have become available in recent years in the form of a WHO pre-qualified oral vaccine and the establishment of international alliances and networks to reduce cholera on a supranational scale. An iterative process between data generation on cholera epidemiology and molecular characteristics, application of novel interventions, and re-evaluation will help maximize the impact of available resources in the resource-limited context of most African countries.

## Supporting Information

S1 Fig Dendrogram of PFGE (*Not*I digestion) patterns for *V*. *cholerae* O1 isolates. The grid on the top left-hand side of the figure indicates “percentage pattern similarity”. Clusters 1 to 6 are indicated by numbers 1 to 6 shown down the left-hand side of the figure.
(PPTX)
