# Supplementary figures and images for: Comparative Characterization of Vibrio cholerae O1 from Five Sub-Saharan African Countries Using Various Phenotypic and Genotypic Techniques
Source: PLoS One. 2015 Nov 25;10(11):e0142989. doi: 10.1371/journal.pone.0142989 (PMC4659613; doi:10.1371/journal.pone.0142989)

## Slide 1
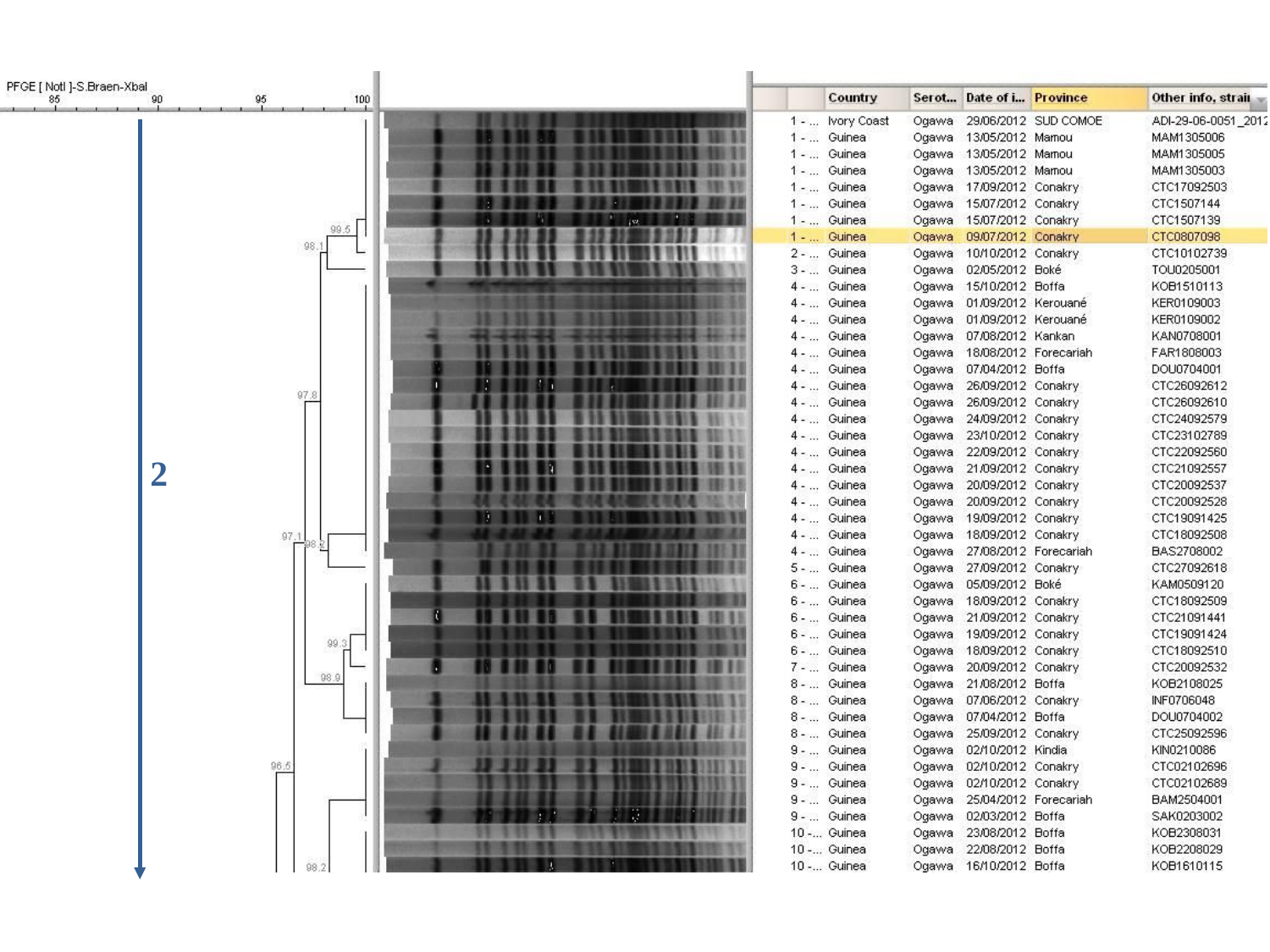

2

## Slide 2
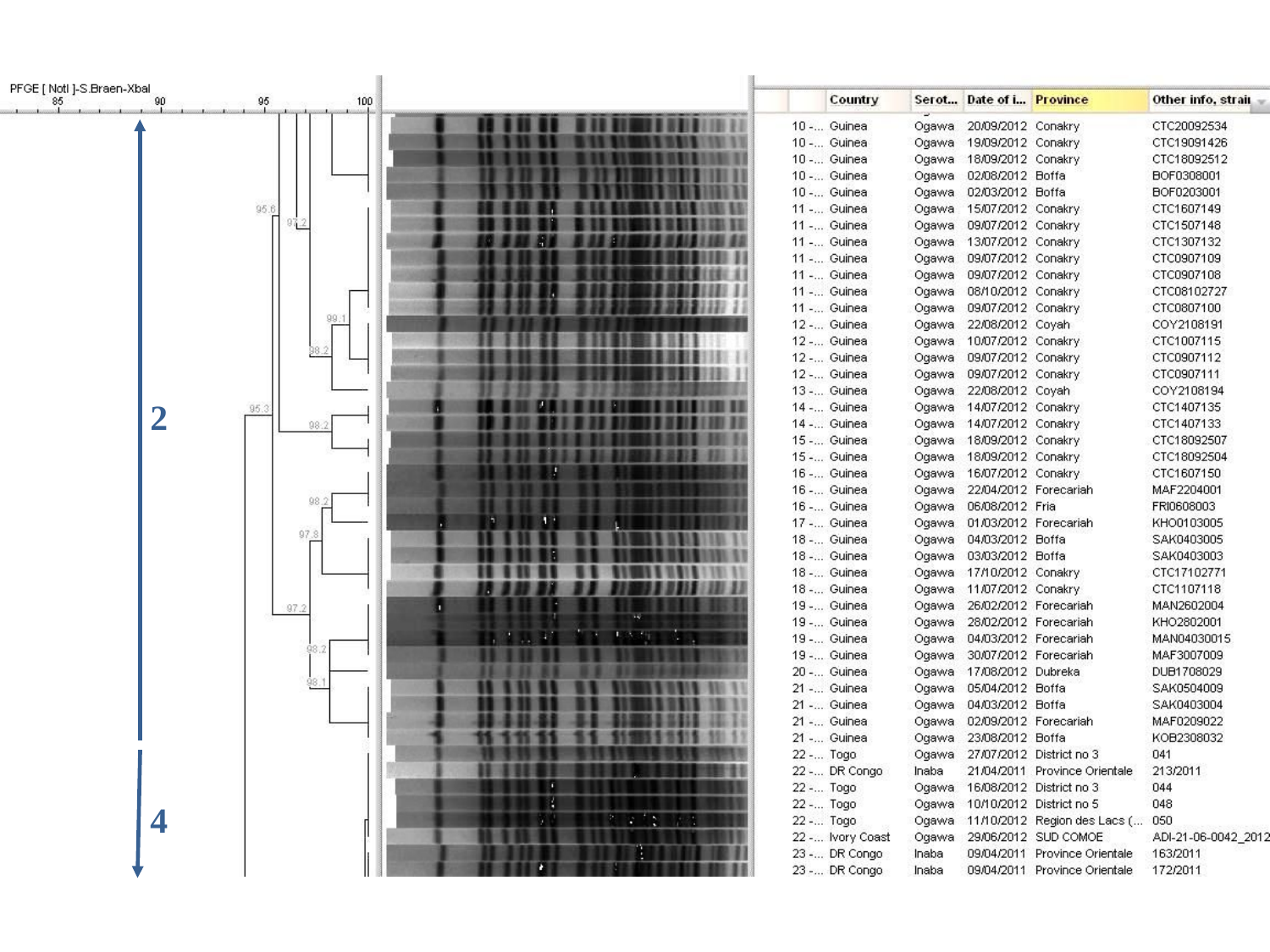

2
4

## Slide 3
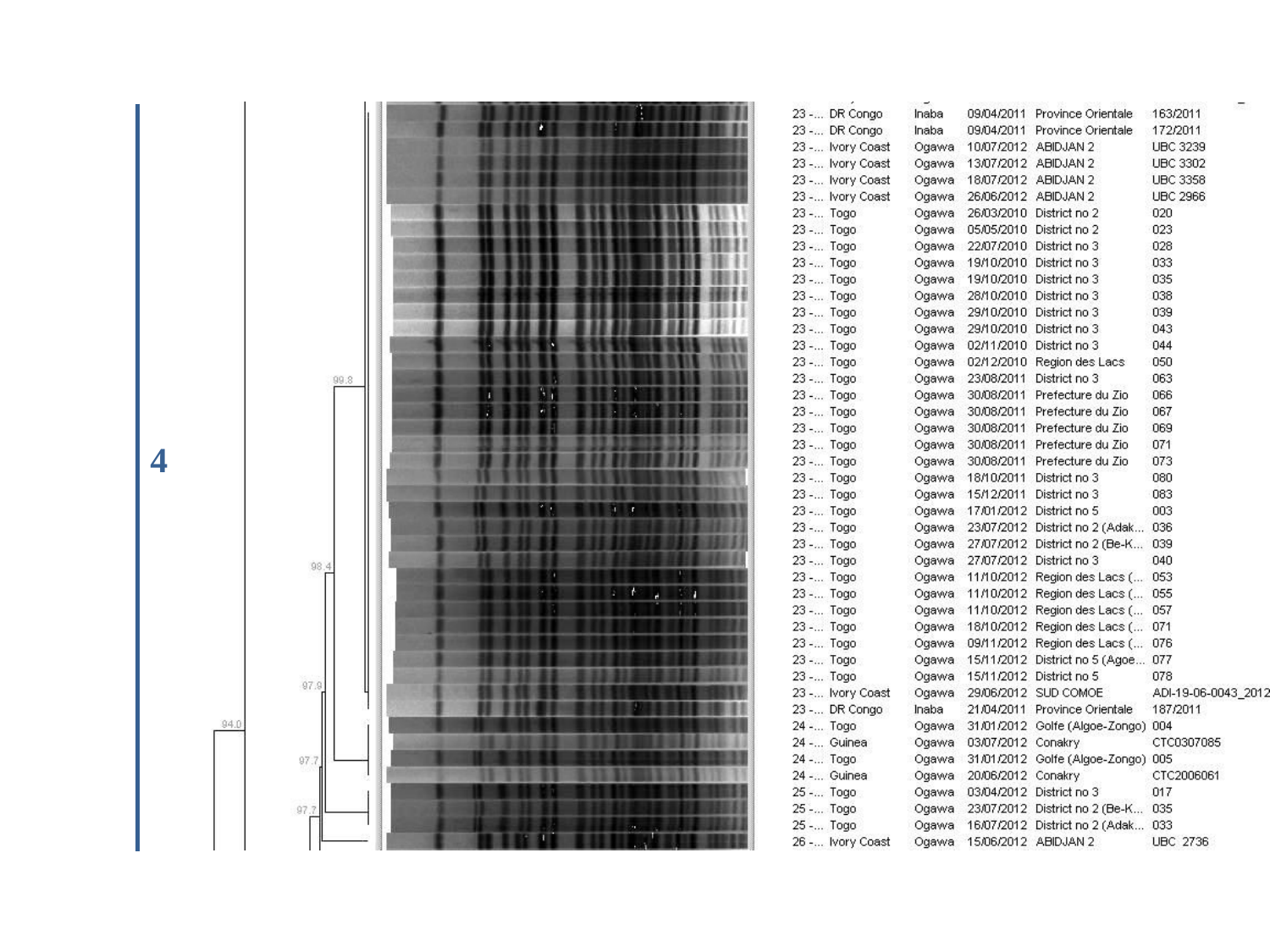

4

## Slide 4
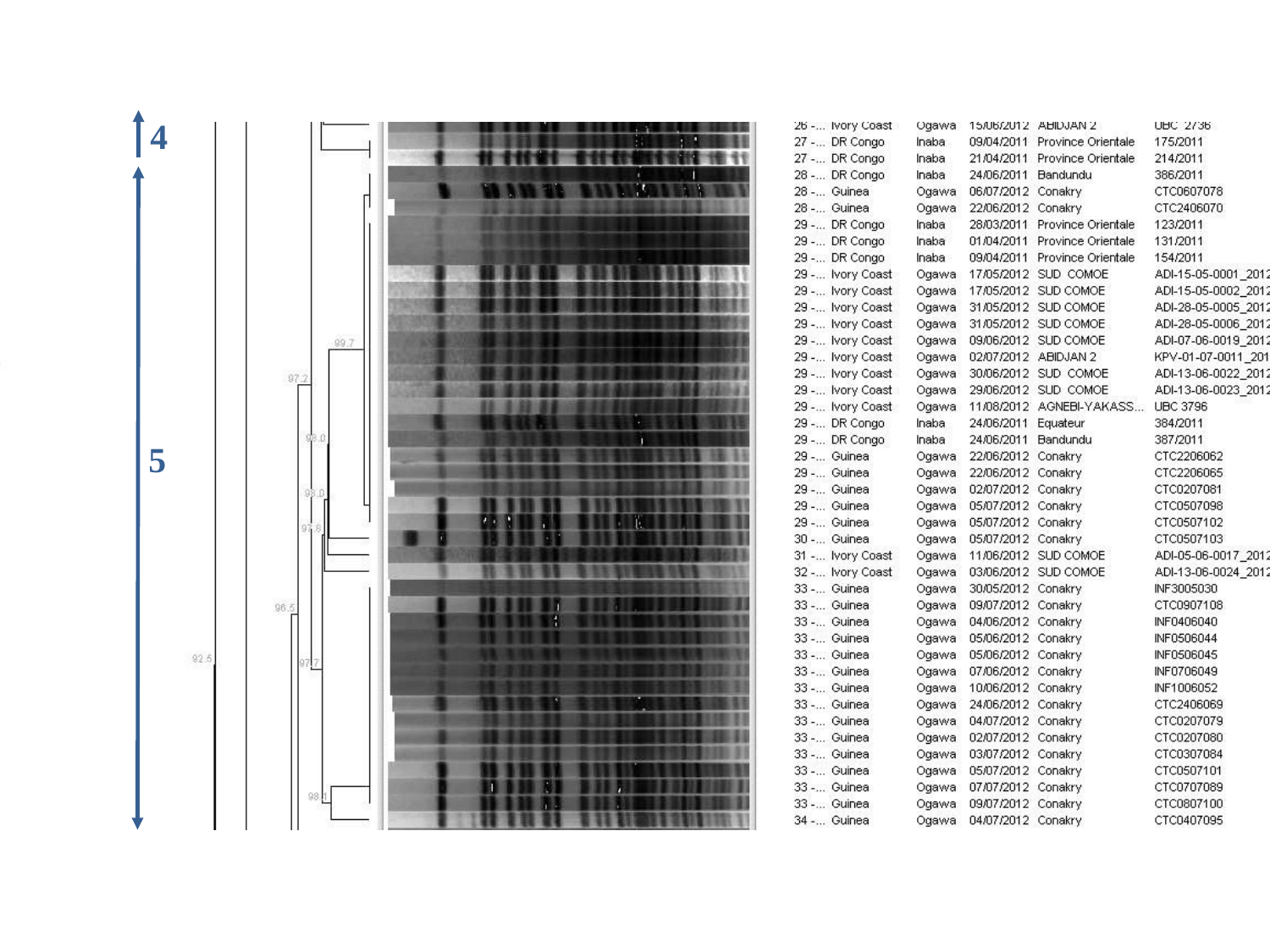

4
5

## Slide 5
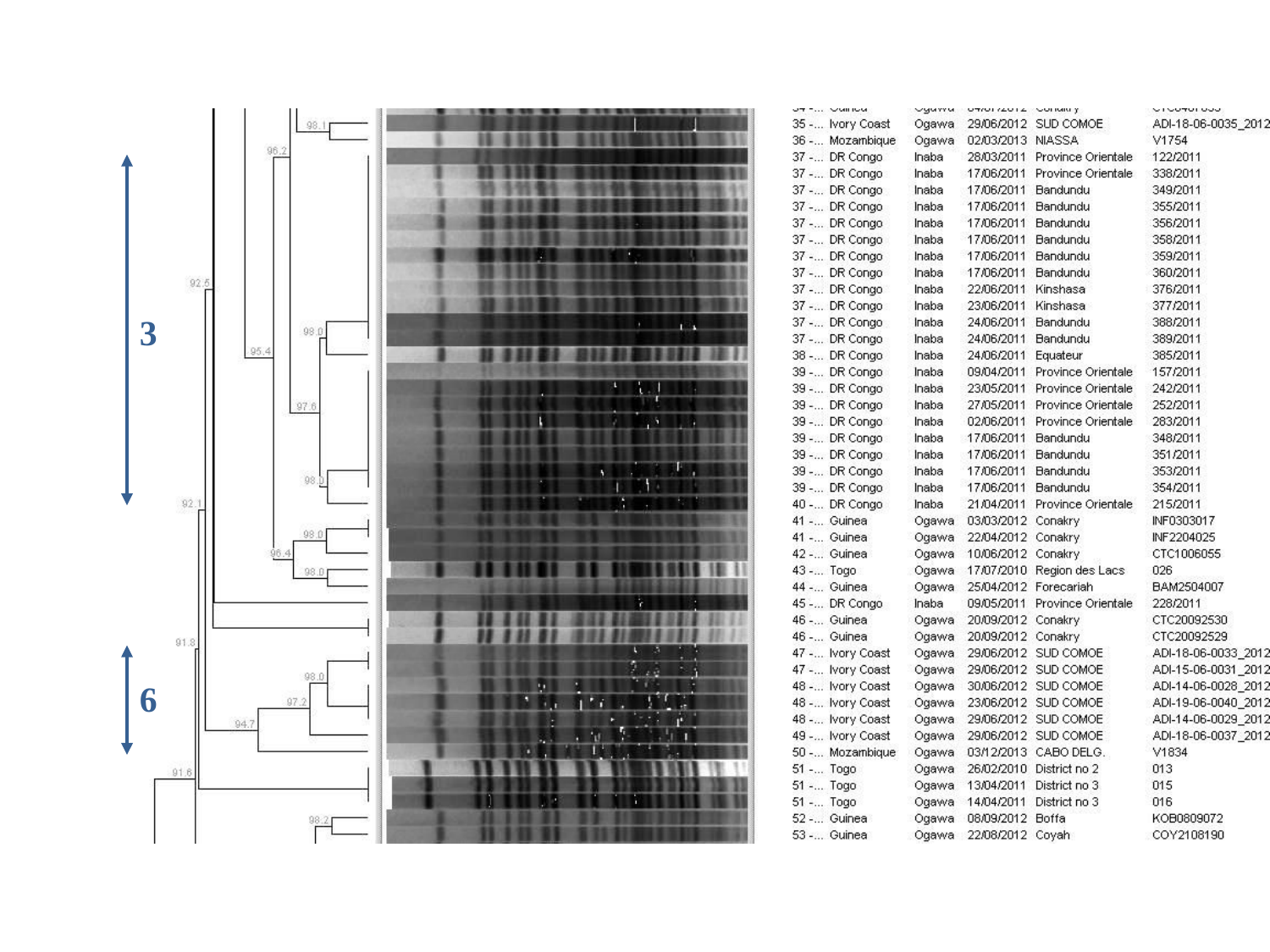

3
6

## Slide 6
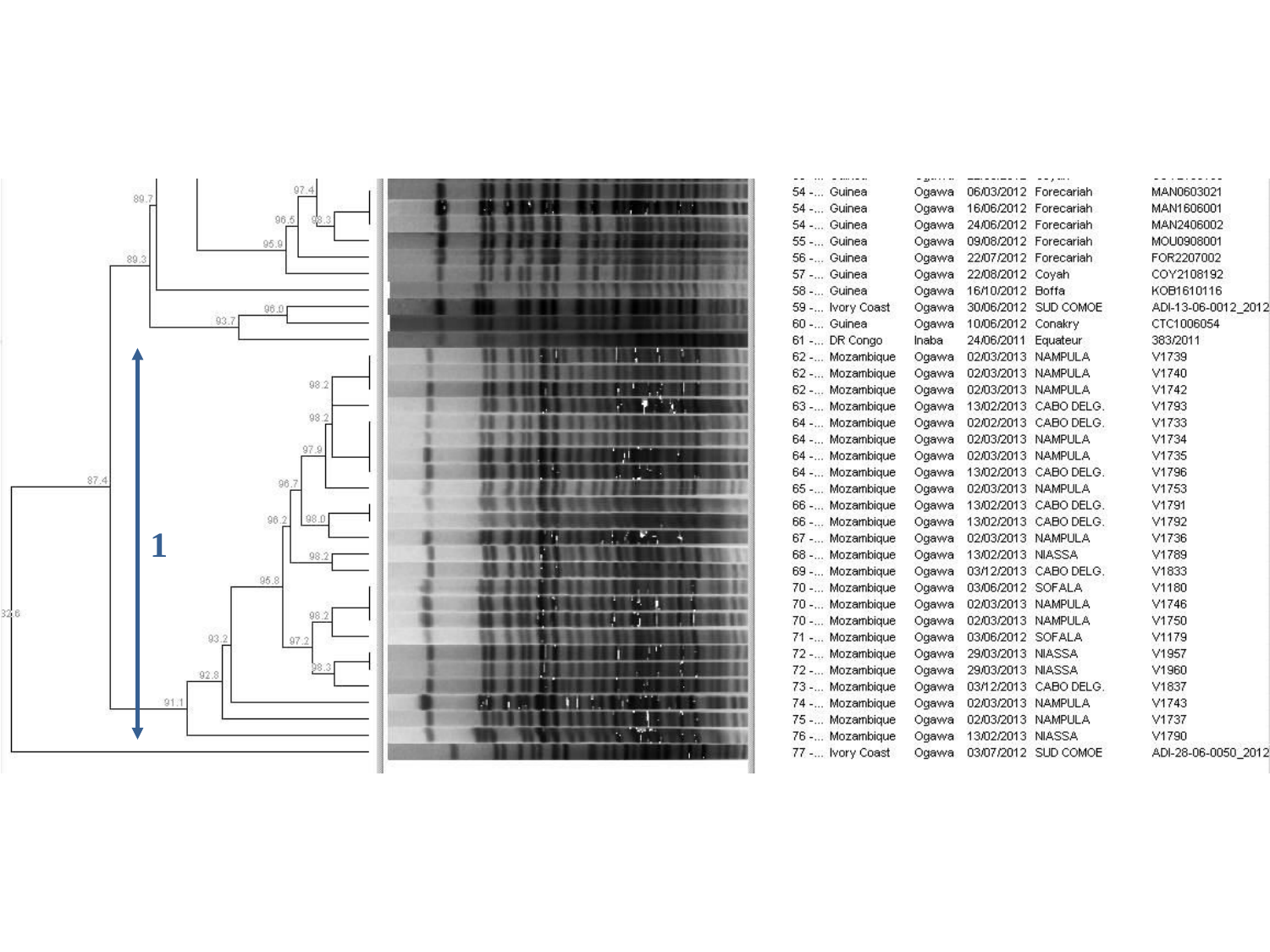

1

Supplement: S1 Fig — The grid on the top left-hand side of the figure indicates “percentage pattern similarity”. Clusters 1 to 6 are indicated by numbers 1 to 6 shown down the left-hand side of the figure. (PPTX) [file pone.0142989.s001.pptx]
